# Supplementary material for: Role of Transporters and Enzymes in Metabolism and Distribution of 4-Chlorokynurenine (AV-101)
Source: Mol Pharm. 2024 Jan 23;21(2):550–63. doi: 10.1021/acs.molpharmaceut.3c00700 (PMC10848289; doi:10.1021/acs.molpharmaceut.3c00700)
Supplement: Supplementary file 1 — mp3c00700_si_001.pdf [file mp3c00700_si_001.pdf]

Role of transporters and enzymes in metabolism and distribution of 4-chlorokynurenine (AV-101)

Running title: Pharmacogenetic predicators of 4-CL-KYN & metabolites concentrations in plasma

Waseema Patel[1], Ravi G. Shankar[2], Mark A. Smith[3,], H. Ralph Snodgrass[4], Munir Pirmohamed[1], Andrea L. Jorgensen[2], Ana Alfirevic[1] & David Dickens[1*]

[1] Department of Pharmacology and Therapeutics, University of Liverpool, Liverpool, L69 3GL UK.

[2] Institute of Population Health, University of Liverpool, Liverpool, L69 3GL, UK.

[3] Vistagen Therapeutics, Inc., 343 Allerton Ave, South San Francisco, California, 94080, USA.

[4] Formerly at Vistagen Therapeutics, Inc., 343 Allerton Ave, South San Francisco, California, 94080, USA.

*Correspondence to: Dr David Dickens
Department of Pharmacology and Therapeutics
University of Liverpool, Liverpool, L69 3GL, UK
Contact: +44 (0)151 795 5391
Email: david.dickens@liverpool.ac.uk

Supporting Information file

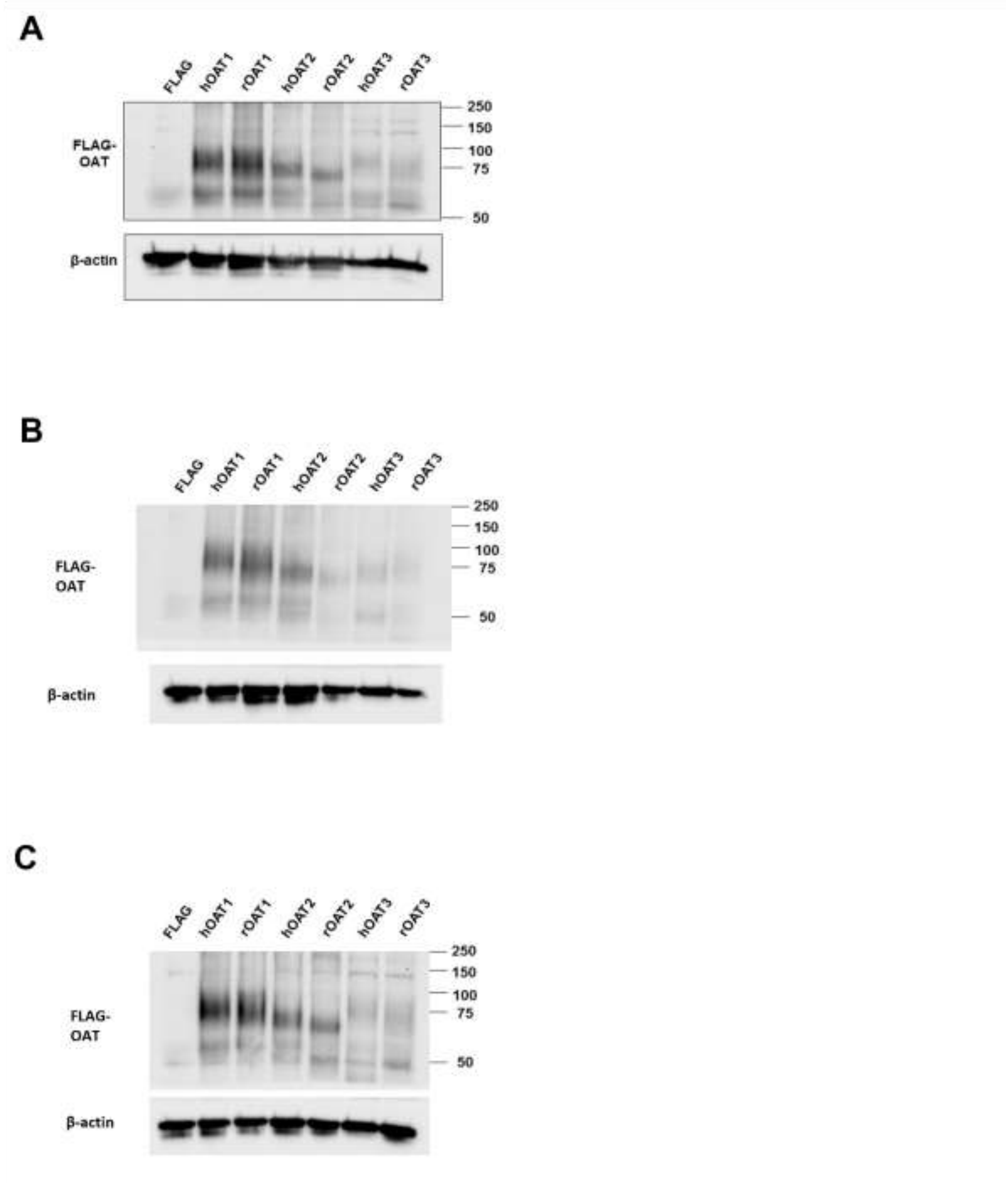

Supplementary Figure 1. Western blots for the expression of human and rat FLAG-OATs transiently transfected into HEK293 cells**.** Three independent repeats for transient transfection with western blotting for OATs tagged to FLAG and β-actin used as a loading control. **(A)** Western repeat 1 **(B)** Western repeat 2 **(C)** Western repeat 3.
